# Disulfiram repurposing combined with nutritional copper supplement as add-on to chemotherapy in recurrent glioblastoma (DIRECT): Study protocol for a randomized controlled trial

**DOI:** 10.12688/f1000research.16786.1

**Published:** 2018-11-15

**Authors:** Asgeir Store Jakola, Katja Werlenius, Munila Mudaisi, Sofia Hylin, Sara Kinhult, Jiri Bartek, Øyvind Salvesen, Sven Magnus Carlsen, Michael Strandéus, Magnus Lindskog, David Löfgren, Bertil Rydenhag, Louise Carstam, Sasha Gulati, Ole Solheim, Jiri Bartek, Tora Solheim

**Affiliations:** 1 Department of Neurosurgery, Sahlgrenska University Hospital, Gothenburg, Sweden; 2 Department of Neurosurgery, St. Olavs Hospital, Trondheim, Norway; 3 Institute of Neuroscience and Physiology, Department of Clinical Neuroscience, University of Gothenburg, Sahlgrenska Academy, Gothenburg, Sweden; 4 Department of Oncology, Sahlgrenska University Hospital, Gothenburg, Sweden; 5 Institute of Clinical Sciences, University of Gothenburg, Sahlgrenska Academy, Gothenburg, Sweden; 6 Department of Oncology, Linköping University Hospital, Linköping, Sweden; 7 Department of Neurology, Karolinska University Hospital, Stockholm, Sweden; 8 Department of Oncology, Skåne University Hospital, Lund, Sweden; 9 Department of Neurosurgery, Karolinska University Hospital, Stockholm, Sweden; 10 Department of Clinical Neuroscience, Karolinska Institute, Stockholm, Sweden; 11 Department of Public Health and Nursing, Norwegian University of Science and Technology, Trondheim, Norway; 12 Department of Cancer Research and Molecular medicine, Norwegian University of Science and Technology, Trondheim, Norway; 13 Department of Endocrinology, St. Olavs Hospital, Trondheim, Norway; 14 Department of Oncology, County Hospital Ryhov, Jönköping, Sweden; 15 Department of Immunology, Genetics and Pathology, Uppsala University, Uppsala, Sweden; 16 Section of Oncology, Akademiska University Hospital, Uppsala, Sweden; 17 Department of Oncology, Örebro University Hospital, Örebro, Sweden; 18 Department of Neuroscience, Norwegian University of Science and Technology, Trondheim, Norway; 19 Department of Medical Biochemistry and Biophysics, Division of Genome Biology, Science for Life Laboratory,, Karolinska Institute, Stockholm, Sweden; 20 Research Center, Danish Cancer Society, Copenhagen, Denmark; 21 European Palliative Care Research Centre (PRC), Department of Cancer Research and Molecular Medicine, Norwegian University of Science and Technology, Trondheim, Norway; 22 Cancer Clinic, St. Olavs Hospital, Trondheim, Norway

**Keywords:** Randomized controlled trial, glioma, glioblastoma, disulfiram, alkylating agents, brain tumor

## Abstract

**Background:** Disulfiram (DSF) is a well-tolerated, inexpensive, generic drug that has been in use to treat alcoholism since the 1950s. There is now independent preclinical data that supports DSF as an anticancer agent, and experimental data suggest that copper may increase its anti-neoplastic properties. There is also some clinical evidence that DSF is a promising anticancer agent in extracranial cancers. In glioblastoma, DSF induced O ^6^-methylguanine methyltransferase (MGMT) inhibition may increase response to alkylating chemotherapy. A recent phase I study demonstrated the safety of DSF in glioblastoma patients when DSF was administered at doses below 500 mg/day together with chemotherapy. We plan to assess the effects of DSF combined with nutritional copper supplement (DSF-Cu) as an adjuvant to alkylating chemotherapy in glioblastoma treatment.

**Methods:** In an academic, industry independent, multicenter, open label randomized controlled phase II/III trial with parallel group design (1:1) we will assess the efficacy and safety of DSF-Cu in glioblastoma treatment. The study will include 142 patients at the time of first recurrence of glioblastoma where salvage therapy with alkylating chemotherapy is planned. Patients will be randomized to treatment with or without DSF-Cu. Primary end-point is survival at 6 months. Secondary end-points are overall survival, progression free survival, quality of life, contrast enhancing tumor volume and safety.

**Discussion:** There is a need to improve the treatment of recurrent glioblastoma. Results from this randomized controlled trial with DSF-Cu in glioblastoma will serve as preliminary evidence of the future role of DSF-Cu in glioblastoma treatment and a basis for design and power estimations of future studies. In this publication we provide rationale for our choices and discuss methodological issues.

**Trial registration: **The study underwent registration in EudraCT 2016-000167-16 (Date: 30.03.2016,) and Clinicaltrials.gov NCT02678975 (Date: 31.01.2016) before initiating the study.

## Introduction

Glioblastoma is a highly malignant brain tumor associated with limited treatment responses and population based series report median survival of only 10 months ^1^. Standard initial treatment is extensive surgical resections followed by postoperative fractionated radiotherapy, and concomitant and adjuvant temozolomide treatment. Unfortunately, initial treatment prolongs survival only modestly and in principle all glioblastomas eventually recur ^2^. Initial responses to chemotherapy are more often seen in patients with O ^6^-methylguanine methyltransferase (MGMT) hypermethylation, and consequently this is an important predictive biomarker ^3^. At time of recurrence there is no international treatment consensus ^4, 5^. A common approach is to reintroduce temozolomide as long as the progression occurs more than 3–6 months after end of the initial treatment. In patients with tumor progression early after primary treatment or seen on ongoing temozolomide therapy, lomustine (also known as CCNU) ^6^ or the combination regimen procarbazine, CCNU and vincristine (PCV) are often the treatment of choice. However, effect of second line treatment is often limited and new improved treatments are therefore imperative.

‘Repurposing’ of drugs for new indications has gained increased interest ^7^. Disulfiram (DSF) is an inexpensive, generic drug that has been used since 1947 to treat alcohol dependency ^8^. When alcohol is avoided, the drug is well tolerated in doses below 500 mg daily even with concomitant administration of temozolomide ^9^.

DSF has demonstrated a wide range of antineoplastic effects, potentially also in gliomas ^10– 16^. The interaction of DSF with copper (DSF-Cu) is likely responsible for the anti-cancer effect where DSF-Cu inhibit so-called glioma initiating cells at very low concentrations ^17^. DSF-Cu may further inhibit the proteasome system ^10, 17, 18^ and potentiate alkylating DNA damage through impaired DNA repair in glioma cells, an effect that may be mediated through inhibition of MGMT ^17, 19^. Finally, a recent paper demonstrated benefit of cancer patients using disulfiram and suggested ditiocarb–copper complex to be the metabolite of disulfiram with anti-cancer effects through effects on protein turnover ^20^. As a consequence, it is now suggested that DSF-Cu should be implemented in clinical trials ^17, 21^.

We seek to study the efficacy and safety of adding DSF combined with nutritional copper supplement (DSF-Cu) to patients with recurrent glioblastomas and concomitant with alkylating chemotherapy. The study is an academic, multicenter, open labeled randomized controlled phase II/III superiority trial with parallel group design. The aim of this paper is to present the intervention, design, endpoint, and power calculations of this study.

## Methods and design

### Study design

The study is a multi-center, open label randomized controlled phase II/III trial with parallel group design. All patients receive alkylating chemotherapy, and in the experimental arm treatment with DSF-Cu is added. Patients will be in the study at a maximum of 24 months or until death.

### Participants, interventions and outcomes

***Study setting*.** The patients will be recruited from and treated at University Hospitals in Norway and Sweden. The study involves eight centers (Gothenburg, Stockholm, Linköping, Lund, Jönköping, Uppsala, Örebro and Trondheim). The study was opened for inclusion January 2017 and per October 14 2018 we have randomized 36 patients.

**Figure 1.**
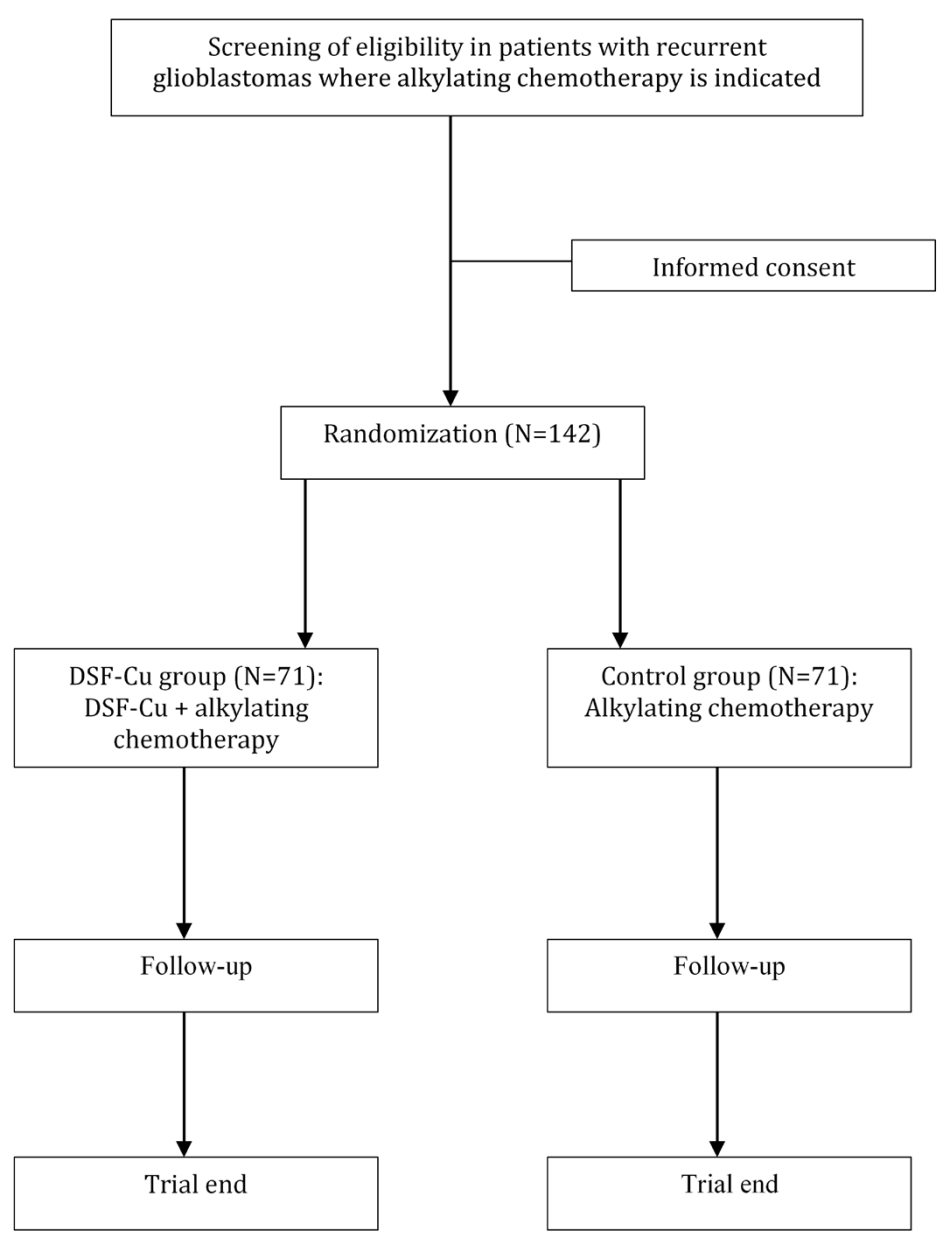
Flow-chart of patient screening, inclusion and follow-up.

***Eligibility criteria*.** Adult patients (≥ 18 years) with first recurrence of glioblastoma will be screened for inclusion from the regular clinical practice at regional oncological departments ( Figure 1). The full eligibility criteria are presented in Table 1. In short, adult patients intended for treatment with alkylating chemotherapy for recurrent glioblastoma are eligible if they have a Karnofsky Performance status 60–100 (ECOG Grade 0-2) ^22^, are willing to refrain from alcohol, and have no major contraindications towards DSF-Cu.

**Table 1.** Full inclusion and exclusion criteria.

Inclusion criteria	Exclusion criteria
Age 18 years or older.	History of idiopathic seizure disorder, psychosis or schizophrenia.
A previous histopathologically verified diagnosis of glioblastoma/ gliosarcoma (later referred to as only glioblastoma) now presenting with a first-time progression/recurrence documented by MRI.	Uncontrolled hypertension (i.e. systolic BP > 180 mmHg) and a diagnosis of congestive heart failure
Planned treatment of recurrence with chemotherapeutic alkylating agents	Received radiotherapy within the 3 months before the diagnosis of tumor progression to reduce risk of misdiagnosis due to pseudoprogression.
Karnofsky performance status of 60 – 100 ^22^.	Addiction to alcohol or drugs.
At inclusion not receiving another experimental treatment, including compassionate use programs, for glioblastoma	Pregnant and/or breastfeeding and women of childbearing potential who do not have a negative pregnancy test taken no longer than 14 days prior to randomization.
Able to take oral medications.	History of liver disease.
No known allergy to DSF or copper.	History of Wilson's disease.
Absolute neutrophil count ≥ 1,500/mcL and platelets ≥ 100,000/mcL	History of hemochromatosis.
Willingness to refrain from ingestion of alcoholic beverages while in the study is a criterion to be randomized. However, once randomized alcohol abstinence only affects the group treated with DSF	Nickel hypersensitivity ^8^
	Need for metronidazole, warfarin and/or theophylline medication since their metabolism may be influenced by DSF.
	Patients who are taking medications metabolized by cytochrome P450 2E1
	Unfit for participation for any other reason judged by the including physician

***Interventions*.** There is no consensus on choice of treatment at first glioblastoma recurrence ^5^. In the Nordic countries, temozolomide, lomustine or PCV are commonly applied. A pragmatic comparator is thus needed to reflect clinical practice. Preclinical data suggest that DSF may enhance the effect of alkylating agents through inhibition of DNA repair ^17^. We therefore use DSF-Cu combination as add-on to “any alkylating chemotherapy” in the intervention group while the comparator group receives “any alkylating chemotherapy” alone.

The administration of DSF and nutritional copper supplement is scheduled to start concomitant with alkylating chemotherapeutic treatment. Patients will take DSF (Antabus ^®^) as a once daily oral dose of 400 mg, based on the known toxicity profile ^9^. In case of intolerance, dose reduction to 200 mg per day is allowed. Copper supplementation will be administered separately from DSF with DSF administration in the evening. Copper nutritional supplement will correspond to 2.5 mg of elementary copper, which is not considered a safety concern ^23^. Adherence to treatment will be assessed at each control with a reminder, self-reporting of drug intake and drug tablet count of remaining DSF. The only surveillance of nutritional copper intake is by self-reporting.

Before study closure no crossover from the control group to DSF-Cu is allowed.

The intervention should continue also after change of chemotherapy, or during temporary chemotherapy withdrawal due to reached maximal cumulative dose or side effects from chemotherapy.

Patients are to discontinue permanently the treatment with DSF-Cu for any of the following reasons:

- Unacceptable adverse effect(s) as judged by the treating doctor.
- No longer indication for tumor targeted therapy.
- The patient wishes to withdraw from treatment.
- If patients develop conditions that contribute to excess risk as judged by the treating physician.

Other experimental therapy, including compassionate use programs, are not allowed in either treatment group as long as the patient is in the trial and receives the assigned treatment.

***Outcomes*.** The primary end-point of the study is survival assessed at 6 months post-randomization.

Secondary end-points are:

- Overall survival from date of randomization.
- Median progression free survival.
- 6 and 12 month progression free survival.
- Health-related generic quality of life ^24^.
- Volumetric growth rate assessed from baseline MRI to first follow-up MRI scan ^25^.
- Safety assessment (toxicities).

***Participant timeline*.** To reduce patient burden, timing of data collection after randomization is scheduled according to choice of chemotherapy regimen, where patients that receive temozolomide usually are assessed every 4 ^th^ week while patients treated with PCV/lomustine are assessed every 6 ^th^ week. Consequently, this may lead to four additional observations in the temozolomide group compared with the lomustine/PCV group. The more detailed clinical control and MRI assessment every 3 ^rd^ month is independent of chemotherapy regimen. The primary endpoint will not be affected by the differences in timing of assessments during the trial.

Finally, hematological status and liver function will be monitored as long as patients are on active treatment every 4–6 weeks, once again depending on the chemotherapeutic protocol ( Table 2).

**Table 2.** Schematic view of study schedule with timing and content of study visits.

Visit	Screening	Monthly	Every 3 months
Month *	0	1,2,4,5,7,8,10,11	3,6,9,12,15,18,21,24
Eligibility	X		
Informed consent	X		
Alcohol screen **	X		X
KPS	X	X	X
Demographics	X		
Pregnancy test	X		
Medical history	X		
Euroqol-5D 3L	X		X
Toxicity/AE/SAE/SAR and SUSAR		X	X
Compliance		X	X
Discontinuation		X	X
Consider drug interactions	X		X
MRI Scan	X		X
Blood samples:			
Hematological and liver monitoring ***	X	X	X

AE denotes adverse event, SAE; serious adverse event, SAR; serious adverse reaction SUSAR; suspected unexpected serious adverse reaction, MRI; magnetic resonance imaging, KPS; Karnofsky Performance Status *The interval for patients treated with lomustine/PCV is 6 weeks, while 4 weeks is clinical routine in the case of temozolomide **Continued alcohol screen only relevant if randomized to the disulfiram group. ***Blood samples as indicated here relevant as long as on active treatment and includes: hemoglobin, thrombocytes, leukocytes with differential count, ASAT, ALAT, G-GT, ALP, bilirubin, INR).

***Sample size and study power*.** The sample size calculation is based on data from previous trials reporting that 50% of patients treated with lomustine deceased within 8 months ^6^. We use a phase III end-point in a phase II setting because this study will be the basis for power calculation of a later more rigid phase III study, and because progression is less relevant in patients with already demonstrated progression/recurrence and with a short expected survival. Due to the phase II/III setting we are relaxing the alpha value to 0.10 (by convention 0.05) since phase II studies are “preliminary assessment of a new intervention before embarking on a larger and expensive randomized controlled trial” ^26^. Thus, we argue that it is good science to slightly increase the chance of false positive results (due to chance) for a relevant end-point instead of using less relevant and difficult to interpret surrogate end-points (such as progression).

Based on the assumption that the treatment group will have an improvement in proportion achieving 6-month survival from 60% to 80% we need a final sample size of 128 in total (64 in each group; alpha 0.1, power 80% and two sided test). Considering 10% attrition, the actual included sample need to be 142 with 71 patients included in each group.

***Assignment of interventions*.** A web-based randomization system (WebCRF 3.0) developed and administered by the Unit of Applied Clinical Research, Faculty of Medicine and Health Sciences, Norwegian University of Science and Technology (NTNU, Trondheim, Norway), will be used. Randomizations are computer generated in a 1:1 ratio with stratification by center. Blocks of varying sizes will be used to make prediction of allocation impossible. Randomization will be performed only after the initial chemotherapy treatment has been selected. Patient enrollment is performed by a study member with appropriate delegation at the local site.

### Data collection, management and analysis

***Data collection*.** Patients will be in the trial for a maximum of 24 months or until death (see study schedule in Table 2). Generic quality of life is followed every 3 ^rd^ month using EQ-5D 3L. Quality of life will be monitored until progression, death or 24 months post-inclusion ^24, 27^.

MRI scanning will be performed at a minimum of every 3 ^rd^ month. MRI assessment may continue up to 24 months post-inclusion and this should be implemented also after documented progression in the DSF-Cu group, as long as the patient continues on only DSF-Cu, or in combination with another established rescue therapy such as surgery, radiation therapy or other alkylating chemotherapy. Consequently, MRI controls are indicated and should be continued as long as tumor targeted therapy is administered (both in control and intervention group). The RANO criteria is used in MRI assessment and for evaluation of progression ^28^.

After withdrawal of tumor target therapy no additional follow-up (including MRI) is planned within the context of this study, in order to minimize patient burden in the end of life setting. Safety assessment are according to the Common Terminology Criteria for Adverse Events (CTCAE) version 4.0 ^29^. Survival is followed until death or 24 months post-inclusion.

***Data management*.** Data will be recorded in the web-based eCRFs (WebCRF 3.0) at The Unit for Applied Clinical Research at the NTNU. All data will be entered in the WebCRF 3.0 at the local study site. All electronic patient information is handled using a study participation number that is not logically connected to any personal data. It is possible to manually “decode” back to personal data using a name list stored securely at the respective study sites.

All data entered into the WebCRF are stored at two separate servers at NTNU. These servers are protected by the general firewall at the university and then by another firewall dedicated to protect clinical research data. The server that stores the data is not the same server as the one in contact with the Internet. There are several further safety measures not to be exposed. There are backups taken every 24-hour throughout the years. The responsibility for this system lies with the Unit for Applied Clinical Research at NTNU.

***Statistical methods*.** All outcomes will be analyzed according to the intention to treat principle unless otherwise specified in post-hoc analyses. This is an open label trial, however the statistician performing analyses will be blinded for treatment assignment. Also, an investigator blinded for treatment allocation will perform the assessment of radiological end-points.

The 6-month survival (i.e primary end-point) will be assessed with 2x2 tables and analyzed with chi-square test. This analysis will be performed 6-months after last patient inclusion. Concerning the primary end-point we do not expect any missing data.

Concerning the secondary outcomes they will be evaluated as follows:

- Actual survival data at 24 months post-inclusion will be analyzed similar to the primary end-point.
- Overall survival analyses will be analyzed using Kaplan-Meier plot and tested with log-rank test. This analysis will be performed at 6 months after the last patient inclusion together with the assessment of primary outcome. Also, this will be analyzed in a longer-term follow-up study (24 months post-inclusion).
- Overall progression free survival will be analyzed with Kaplan-Meier plot and tested with log-rank test. This analysis will also be performed at 6 months and at 24 months after the last patient inclusion. The assessment of progression will be done in accordance with the latest RANO criteria by the local investigator.
- Actual progression free survival analyses at 6 and 24 months after the last patient inclusion. The assessment of progression will be done in accordance with the latest RANO criteria by the local investigator and analyzed similar to the primary end-point.
- Comparison of general health related quality of life at 6 and 24 months after last included patients. We will use an area under the curve approach (i.e. cumulative quality of life) ^27^.
- Semiautomatic segmentation of contrast-enhancing tumor volume assessed at baseline and first MRI control to assess growth dynamics. The data will be analyzed with independent samples t-test or Mann-Whitney U test, based on data distribution.
- Safety assessment with comparing proportion of a composite of grade 3, 4 and 5 toxicities according to the common terminology Criteria for Adverse Events (CTCAE).

We stratify only for study center, however important baseline variables like repeated surgery, time since primary treatment, age, functional status, lomustine versus temozolomide treatment, and biomarker status from initial surgery if available (IDH and MGMT) will be registered and adjusted for in post-hoc exploratory analyses. Also, we will explore outcomes in post-hoc as treated analyses if non-compliance to treatment protocol becomes apparent.

### Monitoring

All source data will be accessible for auditing and monitoring. The external, independent monitor will maintain patient confidentiality. Authorities have the right to perform inspections as well as the monitor.

An initiation visit from study monitors takes place at each site before the enrolment of study subjects at the specific site can start. The first monitoring visit after patient recruitment has started will be performed after inclusion of 5 participants for the specific site, however a minimum of one contact (by e-mail or telephone) and one visit per year to each site is required. After the last study subject has completed the last visit and all CRFs are completed a close-out monitoring visit will take place at each site.

***Harms*.** Adverse events and toxicities is assessed at each study visit and graded according to the NCI Common Toxicity Criteria for adverse events (NCI-CTCAE) Version 4.0 ( http://ctep.cancer.gov/reporting/ctc.html).

***Serious Adverse Event (SAE), Serious Adverse Reaction (SAR) and Suspected Unexpected Serious Adverse Reactions (SUSAR)*.** A serious adverse reaction (SAR) is a serious adverse event (SAE) that may be related to trial treatment. Suspected unexpected serious adverse reactions (SUSAR) are reactions where DSF-Cu may be suspected as the reason for an unexpected SAR. The assessment of relatedness is the responsibility of Department of Clinical Pharmacology at Sahlgrenska University Hospital (Gothenburg, Sweden) *and* the local investigator responsible for the patient.

All reportable cases of adverse events are reported to the Department of Clinical Pharmacology, Sahlgrenska University Hospital. They are responsible to notify sponsor, authorities and local investigators when indicated.

***Interim analysis and criteria for aborting the trial*.** At 50% inclusion (71 included patients) an interim analysis on primary end-point and safety will be performed. An independent data monitoring scientific committee (DMSC) will review the results of this interim analysis. This committee consists of professor Anja Smits (Gothenburg), associate professor Petter Förander (Stockholm) and Dr. Annika Malmström (Linköping). A recommendation signed by all members of the monitoring committee will be handed to the sponsor after this meeting and the final decision is with the sponsor.

Further, the sponsor may terminate the study prematurely for any reasonable cause. The Ethics committees and competent authorities will then be informed. Conditions that may warrant termination include, but are not limited to:

- The discovery of an unexpected, significant, or unacceptable risk to the patients enrolled in the study.
- If competent authorities obtain information that raises doubts about the safety or scientific validity of the study, the competent authorities can suspend or prohibit the study.

### Reporting and transparency

The reporting of this protocol is consistent with the Standard Protocol Items: Recommendations for Interventional Trials (SPIRIT) recommendation ^30^. Reporting of the trial will be consistent with the consolidated standards of reporting trials (CONSORT) 2010 statement ^31^.

### Ethical considerations

This study will be conducted in compliance with the protocol and according to Good Clinical Practice guidelines and applicable regulatory standards. Further, the study will be conducted in accordance with the latest Declaration of Helsinki ^32^. The regional committee in Gothenburg approved the study for all centers in Sweden in a multicenter approval (Dnr 332-16). Ethical approval for the Norwegian site was acquired separately from the ethical committee in region Central Norway (2016/283). Further, the study is approved by the Norwegian medicines agency (16/04133) and the Swedish medical product agency (5.1-2016-25235). Protocol modifications will be reported to the local ethical committees and significant modifications will in addition be communicated to the Norwegian medicines agency and the Swedish medical product agency.

Patients will be included only after the informed consent form is signed ( Supplementary File 1, Swedish version). The informed consent needs to be explicit and written and may at any time be withdrawn by the participant. Also, the right of the participant to refuse to participate without giving reasons must be respected.

Patients without possibility to provide written, informed consent (e.g. severe cognitive deficit and aphasia) are not to be included via proxies.

Patient confidentiality will be maintained through de-identified and secure storage of data and a secure, local storage of the name lists at the respective study sites.

### Dissemination

The principal investigator (ASJ) and the study statistician (ØS) will have access to the final dataset. A cleaned dataset from specific sites may be given back in anonymous form to sites upon request, but only after the final scientific publication (after 24 months follow-up). No other data sharing is planned.

The trial results will be communicated through publication of a scientific report in a peer-reviewed journal. There are no restrictions on publication (e.g. a negative trial will still be published).

### Trial status

At the time of submission the recruitment has not been completed (started January 2017). 

This paper is based on protocol version 2.0, dated December 12 ^th^ 2017.

## Discussion

This study investigates the role of DSF-Cu in recurrent glioblastoma. To our knowledge this is the first planned, randomized controlled trial with DSF-Cu in glioblastoma. The results from this trial will consequently serve as preliminary evidence of the future role of DSF-Cu in glioblastoma treatment as well as to serve as a basis for power estimations in future studies on the same topic.

The study has several pragmatic features, including an open design and a patient centered primary end-point ^33^. Due to the nature of our study end-point, we need to recruit more patients than if we had chosen a surrogate marker which is more common in the phase II setting. Nevertheless, we believe that surrogate markers are less relevant in the setting of glioblastoma recurrence since survival is short and image interpretation and response assessment is difficult ^6, 28^. Consequently, the trial is best described as a phase II/III trial. Further, the trial is to a high degree integrated within the frame of regular clinical practice, reducing the need of extra resources and thereby cost ^33^. The study will thus reflect clinical practice and the little deviation from everyday practice also reduce patient burden.

It may be a personal sacrifice for some patients to abstain from alcohol intake completely. Nevertheless, DSF has a well-known risk profile and the major risks of treatment with 400 mg DSF daily is mainly associated with lack of compliance with respect to alcohol intake and infrequently serious idiosyncratic hepatitis may be seen. In addition, there is a risk of neuropathy that is reversible upon drug discontinuation ^9^. In sum, the risks of DSF-Cu, also combined with chemotherapy, are presumably limited ^9^. The potential benefit lies in improved response to treatment.

The main strengths of this study are the multi-center setting, a hard primary end-point, secondary endpoints relevant to patients, a randomized controlled design and clinically relevant control group representing present best clinical practice. Further, the study being independent from the pharmaceutical industry is another strength. Limitations include the open design making both patients and investigators aware of treatment assignment. Thus, it is difficult to completely guarantee against unscheduled use of DSF-Cu in the control group. To minimize the risk of bias the statistical analyses both for primary and secondary endpoints will be performed by an investigator blinded for the actual treatment. Endpoints will be analyzed before analyzes of adverse events that may reveal actual treatment in the two groups.

In conclusion, we present a protocol for investigating the efficacy of disulfiram and nutritional copper supplement (DSF-Cu) as a supplement to alkylating chemotherapy in the setting of recurrent glioblastoma. We have provided rationale for our choices and discussed methodological issues of importance for this open labeled, randomized controlled trial.

## Data availability

No data is associated with this article.

## Supplementary material

Supplementary File 1: Consent form, in Swedish.
