# Supplementary material for: Disulfiram repurposing combined with nutritional copper supplement as add-on to chemotherapy in recurrent glioblastoma (DIRECT): Study protocol for a randomized controlled trial
Source: F1000Res. 2018 Nov 15;7:1797. [Version 1] doi: 10.12688/f1000research.16786.1 (PMC6325620; doi:10.12688/f1000research.16786.1)
Supplement: Supplementary file 1 [file f1000research-7-18352-s0000.tgz › 1e7d3ed6-be1f-47dd-9cd6-58de1dde2d3c_Supplementary_File_1.pdf]

Titel: Disulfiram som tillägg till kemoterapibehandling vid recidiv av glioblastom; en randomiserad, kontrollerad studie.

Vi tillfrågar dig som behandlas för en hjärntumör om du vill medverka i en forskningsstudie som syftar till att utvärdera effekt och säkerhet av ett läkemedel som heter disulfiram (Antabus®) som tillägg till den vanliga behandlingen bestående av kemoterapi.

Studien bedrivs vid Neurokirurgiska kliniken respektive Onkologiska kliniken på Sahlgrenska Universitetssjukhus samt vid Onkologiska kliniken på Universitetssjukhuset i Linköping, Onkologiska kliniken på Universitetssjukhuset i Lund, Onkologiska kliniken i Uppsala, Onkologiska kliniken i Jönköping, Onkologiska kliniken i Örebro och vid Neurologiska kliniken på Karolinska sjukhuset i Stockholm.

Studien är granskad och godkänd av Regionala etikprövningsnämnden i Göteborg och av Läkemedelsverket.

Bakgrund och syfte

Studien syftar till att utvärdera behandlingseffekten av läkemedlet Antabus® (som innehåller den verksamma substansen disulfiram) hos patienter med hjärntumör. Disulfiram har visat sig ha effekt på cancerceller i laboratorieförsök och i djurmodeller. Man har noterat att effekten tycks öka då det finns en god tillgång till koppar.

Vi vill nu undersöka om tillägg av disulfiram och koppartillskott till rutinmässig cellgiftbehandling ger ett förbättrat behandlingssvar.

Deltagande i studien är helt frivilligt.

Om du väljer att inte delta i studien påverkar detta inte din vård eller relation till din behandlande läkare eller övrig vårdpersonal. Väljer du att delta i studien har du rätt att när som helst och utan närmare förklaring avbryta ditt deltagande. Ett sådant beslut påverkar inte heller ditt övriga omhändertagande på mottagningen eller inom sjukvården. Men även vi kan besluta att avbryta ditt deltagande i studien om det bedöms som nödvändigt för dig eller om du inte uppfyller något av kriterierna i studien. Om det under studiens gång kommer fram ny information som kan påverka studien kommer du att informeras.

Hur går studien till?

Om du bestämmer dig för att vara med i studien, så kommer du att i tillägg till standardbehandlingen bli randomiserad (vilken grupp man kommer i bestäms utifrån ett på förhand uppgjort schema, dvs. studien är randomiserad) till att utöver den vanliga behandlingen även få disulfiram och kosttillskott med koppar. Hälften av personerna som väljer att delta i studien kommer att randomiseras till att få vanlig behandling och hälften till att utöver den få tilläggsbehandling enligt ovan.

Vid ditt första besök kommer vi att göra en extra analys i blod för att kontrollera din kopparomsättning. Det är inget extra blodprov utan analysen görs i ett rutinmässigt blodprov. Detta för att minimera risken för biverkningar.

Som tillägg till vanlig praxis kommer du också att bli tillfrågad om att besvara enstaka livskvalitetundersökningar, kontrollfrågor om bl.a. biverkningar till behandlingen samt kartläggning avseende alkoholvanor. Kvinnor i fertil ålder kommer dessutom att behöva lämna graviditetstest då det inte bedöms säkert att delta i studien under pågående graviditet.

För att ett eventuellt deltagande i studien skall belasta dig så lite som möjligt så kommer uppföljning av studien i möjligaste mån samordnas med övriga kontroller på sjukhuset.

Din medverkan i studien kommer att vara under som mest 2 år. Den aktiva uppföljningen planeras fortsätta så länge du står på behandlingen som du har blivit randomiserad till.

Biobanksprover

Prover som handhas i studien registreras i en administrativ IVO (Inspektionen för Vård och Omsorg) godkänd biobank**.** Samtliga prover kommer att vara kodade, vilket innebär att de inte kan kopplas direkt till dig som person. Kodnyckeln ansvarar forskningsansvarig läkare för och den förvaras oåtkomlig för obehöriga. Proverna kommer endast att användas för de ändamål som angivits ovan. Inga blodprover sparas utan slängs direkt efter analys.

Eventuella biverkningar, risker och obehag

Nackdelarna med att delta i studien inkluderar risk för biverkningar, samt att du inte får inta alkohol under hela behandlingsförloppet om du hamnar i disulfiram-behandlingsgruppen. Intag av alkohol **måste** undvikas under hela behandlingsperioden (alkohol kan tidigast intas en månad efter att behandling med disulfiram är avslutad) pga. reaktioner som man får vid alkoholintag och samtidig disulfirambehandling (t.ex. ansiktsrodnad, galopperande hjärtrytm, och andra hjärtrytmstörningar, pulserande huvudvärk, tung andning, illamående och lågt blodtryck).

Så länge man avstår från alkohol är det vanliga att disulfiram tolereras mycket väl. Dock finns det beskrivna biverkningar. Det finns t.ex. en liten risk för nervpåverkan, leverskada samt psykiska biverkningar (t.ex mani och depression).

Patienter med ökad risk för att få allvarliga biverkningar av disulfiram och kosttillskott kommer inte att erbjudas deltagande i studien. Sannolikheten för att du skall uppleva allvarliga biverkningar av behandling med disulfiram och kosttillskott är därför liten.

För utförlig beskrivning av biverkningar v.g. se bilaga 1.

En del läkemedel kan korsreagera med disulfiram och dessa skall inte tas under studieperioden om du har blivit randomiserad till disulfirambehandling. Din lokala studieläkare ansvarar för detta. Exempel på läkemedel som kan ge oönskad korsreaktion är omeprazol (t.ex. Losec®), warfarin (t.ex. Waran®), samt vissa epilepsimediciner och vissa sorters antibiotika. En mer fullständig förteckning finns i bilagan med möjliga biverkningar (bilaga 1).

Vid alltför hög koncentration koppar i blodet kan man drabbas bland annat kräkningar, buksmärtor och diarré. Risken för att du skall drabbas av för hög kopparkoncentration bedöms som mycket liten med den planerade dosen kosttillskott och ett gott utgångsvärde avseende kopparomsättning.

Finns det några fördelar med att delta?

En möjlig fördel kan vara en bättre respons på behandlingen

Kostnader, Ersättning och Försäkringsskydd.

Att vara med i studien medför inga extra kostnader för dig. Du kommer att få ett recept på disulfiram (Antabus®) att hämta ut på apoteket, och disulfiram omfattas även av högkostnadsskyddet.

Liksom inom sjukvården i övrigt omfattas du av Läkemedelsförsäkringen och Patientförsäkringen för disulfirambehandlingen. För koppar, som är ett kosttillskott och inget läkemedel, finns det tecknat en särskild försäkring. Därmed täcks alla aspekter av den experimentella behandlingen av försäkringen.

Hantering av data och sekretess

Personuppgifter kommer att lagras i en databas och hanteringen av uppgifterna regleras av Personuppgiftslagen (SFS 1998:204). Ditt namn och personnummer kommer att ersättas av en kod, så att inte information om dig som enskild person kan urskiljas. Ingen obehörig har tillgång till databasen. Ändamålet med databasen är forskning. Då forskning betraktas som allmänt intresse så är det den rättsliga grunden för hantering av personuppgifter. Endast de som är ansvariga för studien har tillgång till en identifieringslista som förvaras inlåst. Data som har samlats in sparas i 10 år, för att därefter raderas.

Undersökningsresultat som kommer att presenteras kommer inte att röja enskilda individer.

Enligt Dataskyddsförordningen, GDPR, (EU 2016/679) har du rätt att ansöka om vilka personuppgifter som behandlas, få rättelse eller radering av personuppgifter. Du har också rätt att begära begränsning av behandlingen av dina personuppgifter eller att invända mot behandlingen. Utförarstyrelsen för Sahlgrenska Universitetssjukhuset är ansvarig för behandlingen av personuppgifterna. Dataskyddsombudet är den person som ansvarar för att dina personuppgifter behandlas på ett lagligt och korrekt sätt. Vid behov kan dataskyddsombudet hjälpa till så att du kan få information om vad som registrerats och få eventuella rättelser genomförda. Dataskyddsombudet går att nå på adress: Sahlgrenska Universitetssjukhuset, Dataskyddsombudet, 413 45 Göteborg. Telefon 031-343 27 15.

Om du vill lämna in ett klagomål angående behandlingen av personuppgifter så kan du vända dig till Datainspektionen som är tillsynsmyndighet för dataskyddsförordningen.

Dina personuppgifter kommer endast att användas för de ändamål som angivits ovan. De kan endast komma att behandlas för andra syften om du lämnat ett nytt samtycke och/eller nytt godkännande erhållits av etikprövningsnämnd.

Förutom sjukvårdspersonal kan en person utsedd av kliniken eller en myndighetsperson komma att jämföra insamlade studiedata med din medicinska journal i kvalitetssyfte för att se om studien blivit rätt genomförd. Dessa personer måste underteckna en sekretessförbindelse innan de får tillgång till din medicinska journal. Studiedata sparas minst 10 år efter att studien är avslutad för att möjliggöra kontroller. Genom att du skriver under samtycket ger du din tillåtelse till denna insyn i din patientjournal.

Hur får jag information om mina resultat?

Resultaten kommer att publiceras i vetenskapliga tidskrifter och presenteras på vetenskapliga konferenser. Enbart statistik kommer att presenteras och ingen enskild person kommer att kunna identifieras.

Kontaktpersoner

Huvudansvarig läkare är Asgeir Jakola, MD, PhD

Neurokirurgisk avdelning, Sahlgrenska Universitetssjukhuset, Göteborg, Blå Stråket 5, vån 3, 41345 Göteborg, Sweden. Telefon arbeid: +46 (0)0313429741

Medansvariga läkare i Göteborg är: Katja Werlenius, onkologiska kliniken, Sahlgrenska Universitetssjukhuset, Telefon: +46 (0)313428661

Medansvariga läkare i Linköping är: Munila Mudaisi, Onkologiska kliniken, Universitetssjukhuset i Linköping, Telefon: +46 (0)101038256

Medansvariga läkare i Stockholm är: Sofia Hylin, neurologiska kliniken, Karolinska Universitetssjukhuset, Telefon: +46 (0)851772011

Medansvariga läkare i Lund är: Sara Kinhult, Onkologiska kliniken, Skånes Universitetssjukhus (Lund), Telefon +46 (0) 46134614

Medansvariga läkare i Jönköping är: Michael Strandéus, Länssjukhuset Ryhov, Onkologkliniken, Telefon +46 (0) 36329482.

Medansvariga läkare i Uppsala är: Magnus Lindskog, Onkologiska kliniken, Akademiska Sjukhuset, Telefon +46 (0)186111880.

Medansvariga läkare i Örebro är: David Löfgren, Onkologiska kliniken, Universitetssjukhuset Örebro, Telefon +46 (0)196022162.

Du är alltid välkommen att ringa till oss om du vill fråga något i anslutning till studien.

_____________________________ Telefon: _____________________________

_____________________________ Telefon: _____________________________

Forskningspersonens samtycke

Titel: Disulfiram som tillägg till kemoterapibehandling vid recidiv av glioblastom; en randomiserad, kontrollerad studie.

Jag har muntligen informerats om ovanstående studie och läst bifogad skriftliga information. Jag har fått tillfälle att ställa frågor och fått eventuella frågor besvarade.

Jag känner till att mitt deltagande är helt frivilligt. Jag är medveten om att jag när som helst och utan närmare förklaring kan avbryta mitt deltagande utan att det påverkar mitt framtida omhändertagande.

Jag samtycker till:

- Att deltaga i studien.
- Att mina studiedata (personuppgifter) får behandlas som beskrivits.
- Att mina prover får användas som beskrivits
- Att en central granskare eller en myndighetsperson får jämföra insamlade studiedata med information i min medicinska journal. Detta får ske under förbehåll om sekretess.

______________________ ______________________________________

Datum Forskningspersonens underskrift

____

Forskningspersonens namnförtydligande

Information lämnad av:

______________________ ______________________________________

Datum Namnteckning

____

Namnförtydligande

En kopia av denna signerade information ges till patienten

Bilaga 1.

Eventuella biverkningar och korsreaktioner vid medicinering med disulfiram (Antabus)

Liksom alla läkemedel kan disulfiram (Antabus®) ha biverkningar.
Vanliga (förekommer hos fler än 1 av 100 användare):
Trötthet, sömnighet, magbesvär, diarré, dålig andedräkt och metallsmak. Huvudvärk. Mani. Nedstämdhet.
Mindre vanliga (förekommer hos mellan 1 av 100 och 1 av 1000 användare):
Acneliknande utslag eller allergiska utslag med klåda och/eller rodnad. Störd sexualfunktion.
Sällsynta (förekommer hos färre än 1 av 1000 användare):
Stickningar, domningar och muskelsvaghet i framför allt armar och ben. Nedsatt syn och värk i och kring ögat. Förändrad blodbild. Allvarliga leverfunktionsstörningar, ibland med gulsot. Psykiska störningar i form av bla personlighetsförändring.

Användning av andra läkemedel

Följande läkemedel kan påverka eller påverkas av behandling med disulfiram (Antabus):

omeprazol (ex: Losec®), metronidazol (t.ex. Flagyl®), warfarin (t.ex. Waran®), teofyllin, fenytoin (t.ex. Epanutin®, Fenantoin Recip®), fenobarbital, chlordiazepoxid, imipramin, diazepam (t.ex. Stesolid®), isoniazid eller amitriptylin

Alla former av alkohol ska undvikas, även de små mängder som kan förekomma i vissa födoämnen och läkemedel.

Försiktighet skall iakttas med disulfiram (Antabus®)

Om du får symtom såsom aptitlöshet, trötthet, illamående, kräkningar, svaghet, feber, klåda, gulfärgade ögonvitor, gulfärgad hud, mörkfärgad urin eller ljus avföring ska du kontakta din studieläkare. Symtomen kan bero på en leverinflammation.
