# Supplementary material for: Disulfiram repurposing combined with nutritional copper supplement as add-on to chemotherapy in recurrent glioblastoma (DIRECT): Study protocol for a randomized controlled trial
Source: F1000Res. 2018 Nov 15;7:1797. [Version 1] doi: 10.12688/f1000research.16786.1 (PMC6325620; doi:10.12688/f1000research.16786.1)
Supplement: Peer review history

# Peer review: Referee response for version 1

**DOI:** 10.5256/f1000research.18352.r42298

**Published:** 2019-01-03

**Authors:** Frantz R. Poulsen

**Affiliations:** 1 Department of Clinical Research, University of Southern Denmark, Odense, Denmark

The study protocol describes a randomized controlled trial investigating if addition of disulfiram and copper to the standard Stupp regimen for treatment of glioblastoma will increase survival at 6 months, overall survival, progression free survival etc.The study is limited to patients with first recurrence.

 The study is well designed and well planned to answer the research questions. It is, however, more and more common practice to perform surgical resection of the recurrence (if possible) prior to additional chemo therapy. I cannot from the study protocol read if this is also the case here and how the authors will deal with this.

 The RANO criteria will be used in the assessment of progression. Although these criteria are well recognized they may not always be that easy to use and conclude from. Therefore it was very nice, the authors also use volumetric measurements to evaluate growth rate. The authors might, however, consider using other measures than contrast enhancement to assess volumetric growth rate (flair, T2 etc).

I have read this submission. I believe that I have an appropriate level of expertise to confirm that it is of an acceptable scientific standard.

# Peer review: Referee response for version 1

**DOI:** 10.5256/f1000research.18352.r40689

**Published:** 2018-11-23

**Authors:** Kalkunte S. Srivenugopal

**Affiliations:** 1 Department of Pharmaceutical Sciences, School of Pharmacy, Texas Tech University Health Sciences Center, Amarillo, TX, USA

The alcohol aversion and metal-chelating drug disulfiram (DSF) exhibits strong affinity for cysteines present in various regulatory proteins. The irreversible mixed disulfides thus formed inactivate the biological activities of several signaling, DNA repair, and anticancer targets, through the same mechanism by which the drug inhibits ALDH and prevents binge drinking. The strong anticancer effects of DSF particularly in combination with copper against gliomas, its long-term use, safety and tolerability, penetration through the blood brain barrier have made it attractive to repurpose the drug for brain cancers. DSF’s ability to Inhibit MGMT, the single most important determinant of glioma resistance to alkylating agents is an additional feature that can increase the efficacy of temozolomide in the trial.

 The current protocol to evaluate the use Cu-DSF in a non-alcoholic setting for recurrent glioblastoma is well designed and well justified. First, there is no consensus on treatment for GBM recurrence and the high-risk patient selection is appropriate. The therapeutic outcome between the two arms (Cu-DSF with or without alkylators) should be well-comparable. Whether the Cu-DSF by itself will have anti-glioma effects in a clinical setting is not known and difficult to predict. This is because, the copper chelated with DSF [BisN, N-diethyldithiocarmato) copper II], essentially appears to be an inactivated molecule and lacks the ability to form S-conjugates and fails to inhibit proteasome and other targets. The fate of  DDC-Cu and its cytotoxic mechanisms *in vivo* are still unclear. Other comments and suggestions are listed below.

1. Whether the protocol uses copper gluconate or another chelated form is not mentioned. There is one study (Proc Amer Assoc Cancer Res. 58, Abstract 4053, 2017) which describes that the efficacy of DSF-Cu is highly dependent on the time (at least 6 h) and schedule (DSF prior to copper) in brain tumor cell cultures. 
2. There is a good chance of myelosuppression, particularly, in patients taking DSF-Cu and temozolomide or other alkylating agents. Therefore, blood cell counts more frequently than 4-6 weeks is suggested. 
3. Cu-DSF is likely to sensitize GBMs to radiation as well. If there are a subset of patients getting radiation alone or in combination with DSF-Cu, it is recommended that the outcomes be assessed as well.

I have read this submission. I believe that I have an appropriate level of expertise to confirm that it is of an acceptable scientific standard.
